# Surgical Management and Predictors of Postoperative Complications of Retrosternal Goiters: A Retrospective Study

**DOI:** 10.7759/cureus.56573

**Published:** 2024-03-20

**Authors:** Yasser A Obadiel, Mohammed Al-Shehari, Yaseen Algmaly, Bilquis Al-Jammra, Iman Kahtan, Nashwan Tashan, Faisal Ahmed

**Affiliations:** 1 General Surgery, Al-Thawra Modern General Hospital, Sana'a, YEM; 2 General Surgery, Faculty of Medicine and Health, Sana'a University, Sana'a, YEM; 3 Surgery, Al-Thawra Modern General Hospital, Sana'a, YEM; 4 Surgery, Faculty of Medicine and Health, Sana'a University, Sana'a, YEM; 5 Surgery, Al-Kuwait University Hospital, Sana'a, YEM; 6 Urology, Ibb University, Ibb, YEM

**Keywords:** surgery, complication, outcome, surgical intervention, thyroid gland, retrosternal goiter

## Abstract

Background: The preferred standard treatment for retrosternal goiter (RSG), a slow-growing, often benign tumor, remains thyroidectomy. An alternative strategy may be required when the goiter is intrathoracic. Data on the results of RSG procedures are rarely reported. Careful patient selection and assessment are critical to avoiding an unexpected sternotomy during surgery and postoperative complications. This study aims to examine the clinical findings and treatment outcomes of RSG and to identify the variables affecting postoperative complications in a resource-limited setting.

Method: A retrospective study was conducted at Al-Thawra Modern General Hospital in Sana'a, Yemen, on 69 patients diagnosed with RSG and undergoing thyroidectomy between April 2019 and February 2023. Initial clinical characteristics, radiological and laboratory findings, treatment approach, and outcome were collected from the patient's medical profile and analyzed. To determine the variables influencing postoperative complications, a bivariate analysis was carried out.

Results: The mean age was 51.0 ± 13.6 years, and 45 (65.2%) were female. The most commonly reported symptoms were palpable masses (66; 95.7%), difficulty breathing (45; 65.2%), and neck discomfort (20; 29.0%), with 7 (10.1%) patients being asymptomatic. Previous thyroid surgery was reported in 10 (14.5%) cases. According to the grading classification, grade 1 was the most prevalent (42; 60.9%). Total thyroidectomy was the predominant surgical procedure in 59 (85.5%) cases. Using a cervical approach, all patients underwent thyroidectomy, and a sternotomy was required in one case. Histopathological analysis revealed benign multinodular goiter in 79.7%, followed by papillary thyroid cancer in 10.1% and thyroiditis in 6.7%. The postoperative complication occurred in 22 (31.9%), and the most common complication was transient hypocalcemia (11, 15.9%). There was no mortality during or after the surgery. In bivariate analysis, advanced age, difficulty swallowing, tracheal deviation, large RSG mass, advanced RSG grade, previous surgery, and malignant histopathology were associated with postoperative complications and were statistically significant (all p<0.05).

Conclusion: RSG is a rare disease that may require challenging surgical intervention. In this study, the cervical approach was the most practical and least intrusive surgical method. In addition, postoperative complications were associated with advanced age, difficulty swallowing, tracheal deviation, large RSG mass, advanced RSG grade, previous surgery, and malignant histopathology. Low postoperative complication rates can be achieved by understanding the surgical architecture of the neck, essential clinical RSG presentation, thyroid pathology, and necessary surgical treatment.

## Introduction

Retrosternal goiter (RSG) is a thyroid mass that invades the thoracic cavity or has more than half of its volume below the thoracic inlet [1]. These RSGs are typically larger and manifest clinically with symptoms related to the proximity of the substernal portion of the gland to the surrounding visceral and circulatory organs [2]. The prevalence of RSG in individuals after thyroidectomy varies considerably due to the lack of established criteria. The incidence of RSG in thyroidectomy patients has been reported to range from 2% to 26% [1]. Although RSG occurs for a long time without symptoms, it can pose a life-threatening risk of sudden growth and compression of the external airway due to complications such as bleeding or malignant transformation [3,4]. Subsequently, surgeons dealing with thyroid diseases face the challenge of assessing acceptable surgical rationale and performing safe, minimally invasive surgeries [5,6].

The selection of patients with RSG for thyroidectomy is a controversial issue. Some argue that only patients with symptoms or suspected cancer should undergo thyroidectomy, while surgeons consider intrathoracic extension an absolute indication for thyroidectomy [5,6]. Various surgical approaches are available for RSG treatment, including cervical incisions, combined thoracic incisions, or thoracic midline incisions, but in most cases, a cervical approach alone can usually be sufficient [2,7]. Reoperation, the size of the thoracic goiter, and intrathoracic protrusion are factors that may influence the outcome. In general, intraoperative and postoperative complications are more common in RSG surgery [2,8].

There is limited information on the extent and features of RSG in resource-limited regions, including Yemen [9,10]. A study by Ghabisha et al. found advanced age, greater thyroid mass, intrathoracic protrusion (higher grade), longer operation time, bleeding, ICU admission, and malignant features associated with postoperative complications [2]. This study aims to examine the clinical presentation and treatment outcome of RSG and to identify the variables affecting postoperative complications in a resource-limited setting.

## Materials and methods

A retrospective study of 69 cases diagnosed with RSG who underwent thyroidectomy between April 2019 and February 2023 was conducted at Al-Thawra Modern General Hospital in Sana'a, Yemen. All medical records of the patients who underwent surgery on RSG patients were reviewed and various factors were taken into account, including demographic characteristics such as age and gender, history of thyroid surgery, clinical presentation (asymptomatic, palpable mass during examination, neck discomfort, difficulty swallowing, difficulty breathing, and dysphonia), radiological imaging (ultrasound, radioactive iodine examination, CT, and MRI), and fine-needle aspiration cytology (FNAC; ultrasound-guided and clinical) results, RSG classification, operative details (time and bleeding), surgical approaches (cervical, total sternotomy, and manubriotomy), type of excision (total thyroidectomy, hemithyroidectomy, and subtotal thyroidectomy), ICU admission, postoperative complications, length of hospital stay, and clinical follow-up were recorded. All patients underwent a standard examination, and surgical procedures were performed by a qualified surgical team. Outcomes included hospital and ICU, length of hospital stay, postoperative complications, and mortality (30 days and at follow-up). The secondary outcome was to determine the risk factors for complications after surgery.

The goiter was diagnosed as RSG after a preoperative CT scan showed that it extended beyond the thoracic inlet to the chest and spanned 50% of the thyroid mass [11]. RSG patients were categorized in this study using the Huins scoring and anatomical categorization system. This categorization system divided goiter disease into three categories: grade 1 (above T4), grade 2 (aortic arch to pericardium), and grade 3 (below right atrium) [11].

Surgical procedures

All procedures were carried out by a single surgeon (Yasser Obadiel), utilizing a normal collar incision and a cervical approach. A supplementary sternotomy was performed only when it was deemed required during the procedure. The recurrent laryngeal nerves were not systematically sought for, but they were usually detected in the last 1-2 cm before entering the larynx.

Statistical analysis

Frequencies and percentages were used to describe the categorical variables and means and standard deviations (SD) were used to describe the numerical variables. The Smirnov-Kolmogorov test was used to determine normality. Depending on the normality of the data, the Mann-Whitney U test or independent samples t-test was used to compare quantitative variables. The significance of the variables' correlations with postoperative complications in the study population was determined using Fisher's exact test or chi-square analysis to examine the associations between qualitative characteristics and postoperative problems. A value that was less than 0.05 was defined as a statistically significant p-value. SPSS Statistics version 22 (IBM Corp. Released 2013. IBM SPSS Statistics for Windows, Version 22.0. Armonk, NY: IBM Corp.) was used for statistical analysis in the study.

Ethical approval

The study was approved by the Ethics Research Committees of Al-Thawra Modern General Hospital (approval number: 2-2024) in compliance with the ethical standards outlined in the Declaration of Helsinki. Due to the anonymous retrospective nature of the study, written informed consent from the included patients was not required.

## Results

The mean age was 51.0 ±13.6 years (range: 24-70 years), and 45 (65.2%) were female. Patient demographic characteristics of RSG are shown in Table 1. The main symptoms at presentation were a palpable mass on examination in 66 (95.7%), difficulty breathing in 45 (65.2%), neck discomfort in 20 (29.0%), difficulty swallowing in 19 (27.5%), and dysphonia in 10 (14.5%) cases, while seven (10.1%) cases were asymptomatic. The duration of symptoms was seven years (range: five months to 25 years). Ten (14.5%) cases had a prior thyroidectomy.

**Table 1:** Demographic characteristics of the patient with RSG RSG: retrosternal goiter

Variables	N (%)
Age (year), mean ± SD	51.0 ± 13.6 (range 24-70)
Sex	
Male	24 (34.8%)
Female	45 (65.2%)
Symptoms	
Palpable mass	66 (95.7%)
Breathing difficulty	45 (65.2%)
Neck discomfort	20 (29.0%)
Swallowing difficulty	19 (27.5%)
Dysphonia	10 (14.5%)
Asymptomatic	7 (10.1%)
Previous operation	10 (14.5%)

All patients were euthyroid upon admission for surgery. Chest radiographs showed mediastinal opacification in 45 patients (65.2%) and tracheal deviation with compression in 30 patients (43.5%).

**Figure 1:**
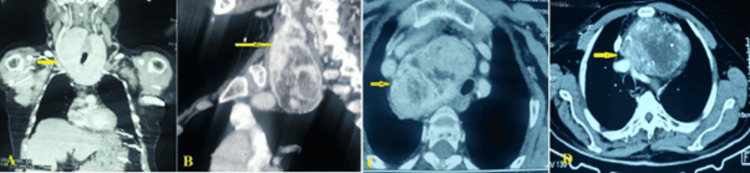
CT scan showing retrosternal thyroid mass (A) coronal view (arrow), (B) lateral view (arrow), (C) mass causing tracheal deviation (arrow), and (D) big thyroid mass with calcifications (arrow) CT: computed tomography

Ultrasound examination confirmed the substernal form of the goiter in all cases. Regarding the classification type of RSG, 42 (60.9%) cases were grade 1 (above the aortic arch (above T4)) and 27 (39.1%) cases were grade 2 (aortic arch to pericardium). In contrast, no patient had a grade 3 RSG (below the right atrium) (Figure 1).

**Figure 2:**
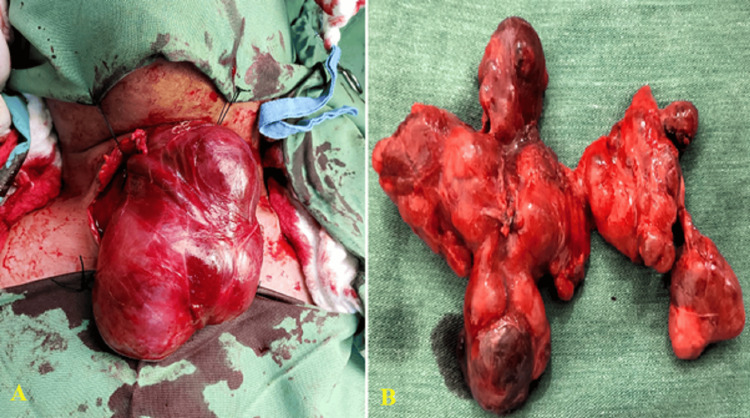
Intraoperative photos showing (A) cervical approach and (B) big thyroid mass in a 44-year-old male with a weight of 600 grams, with a final pathology of goiter

All patients were operated on under general anesthesia by an experienced general surgeon (Figure 2).

All patients underwent retrosternal thyroidectomy via a cervical approach, and in one case, a sternotomy was required. Surgery included total thyroidectomy in 59 (85.5%) cases, hemithyroidectomy in seven (10.1%) cases, and completion thyroidectomy in three (4.3%) cases. Histopathological analysis revealed multinodular goiter in 79.7% of cases, papillary carcinomas in seven (10.1%) (of which two were associated with adenopathy on admission), and thyroiditis in seven (10.1%). Malignancy was not diagnosed preoperatively because it was difficult to perform fine-needle aspiration for RSG, and there was no family history and no biological assessment. The mean weight of the resected mass was 172 ± 68.9 grams (median: 145 grams; min: 100, max: 600). The mass size was between 5.3 cm and 15 cm. Thirty-five (50.7%) required ICU admission. The postoperative complications occurred in 22 (31.9%). Transient hypocalcemia in 11 (15.9%), permanent hypocalcemia in two (2.9%), transient hypercalcemia in two (2.9%), transient hoarseness in eight (11.6%), postoperative hematoma in two (2.9%), local infection in one (1.4%), and seroma collection in one (1.4%) case (Table 2).

**Table 2:** Summary of preoperative, operative, and postoperative results *Some cases had multiple postoperative complications ICU: intensive care unit

Variables	N (%)
Retrosternal grade	
Grade 1	42 (60.9%)
Grade 2	27 (39.1%)
Grade 3	0 (0.0%)
Thyroid weight (ml), mean ± SD	172 ± 68.9 grams
Operative time (min), mean ± SD	89.0 ± 21.3 (range: 56.0-166 min)
Types of operations	
Completion thyroidectomy	3 (4.3%)
Hemi-thyroidectomy	7 (10.1%)
Total thyroidectomy	59 (85.5%)
Need sternotomy	1 (1.4%)
Need ICU	35 (50.7%)
Postoperative complication*	22 (31.9%)
Transient hypocalcemia	11 (15.9%)
Permanent hypocalcemia	2 (2.9%)
Transient dyspnea	2 (2.9%)
Postoperative hematoma	2 (2.9%)
Local infection	1 (1.4%)
Seroma collection	1 (1.4%)
Transient hoarseness	8 (11.6%)
Histopathologic findings	
Multi-nodular goiter	55 (79.7%)
Papillary thyroid carcinoma	7 (10.1%)
Thyroiditis	7 (10.1%)

Factors associated with postoperative complications

In bivariate analysis, advanced age, difficulty swallowing, tracheal deviation, large RSG mass, advanced retrosternal grade, previous surgery, and malignant histopathology were associated with postoperative complications and were statistically significant (all p<0.05) (Table 3).

**Table 3:** Bivariate analysis of risk factors for postoperative complications of total thyroidectomy for substernal goiter Note: Boldface indicates a statistically significant result (p<0.05)

Characteristic	Without complication N = 47 (68%)	With complication N = 22 (32%)	p-value
Age (year)	44 (34, 55)	65 (62, 67)	<0.001
sex			0.37
Male	18 (38%)	6 (27%)	
Female	29 (62%)	16 (73%)	
Symptomatic	43 (91%)	19 (86%)	0.67
Palpable mass during an examination	44 (94%)	22 (100%)	0.55
Neck discomfort	12 (26%)	8 (36%)	0.36
Swallowing difficulty	8 (17%)	11 (50%)	0.004
Breathing difficulty	28 (60%)	17 (77%)	0.15
Dysphonia	4 (8.5%)	6 (27%)	0.064
Tracheal deviation	13 (28%)	17 (77%)	<0.001
Size of thyroid mass	134 (122, 150)	220 (210, 230)	<0.001
Retrosternal grade			<0.001
Grade 1	40 (85%)	2 (9.1%)	
Grade 2	7 (15%)	20 (91%)	
Previous operation	1 (2.1%)	9 (41%)	<0.001
Pathology result			<0.001
Multi-nodular goiter	47 (100%)	8 (36%)	
Papillary thyroid carcinoma	0 (0%)	7 (32%)	
Thyroiditis	0 (0%)	7 (32%)	

## Discussion

In this study, we retrospectively reported our experience in the surgical management of RSG using the cervical approach and investigated the predictive factors for postoperative complications in a resource-limited setting. Goiter is a complex disease characterized by the growth of the thyroid gland as an adaptive response to reduced production of thyroid hormones. The main causes include inflammatory diseases such as Hashimoto's thyroiditis, infiltrative diseases such as sarcoidosis, and postpartum thyroiditis in wealthy countries, while iodine deficiency is the most common cause of goiter in low-income countries and worldwide [12]. The exact cause of RSGs is uncertain. It appears to be a combination of factors, including a low larynx, goiter enlargement, gravity, negative intrathoracic pressure, and rotation of the larynx during swallowing [7].

The manifestation of a substernal goiter can range from asymptomatic or mild symptoms to obstructive or compressive symptoms during swallowing or breathing, resulting in significant impairment [13]. Because a goiter develops slowly, those affected may be asymptomatic despite significant tracheal narrowing or esophageal deformity and only realize how narrow they are after the goiter has been removed [7]. In this study, the most commonly reported symptoms were palpable masses (66; 95.7%), difficulty breathing (45; 65.2%), and neck discomfort (20; 29.0%), with seven (10.1%) patients being asymptomatic. In Abdelrahman et al.'s study, dyspnea (40%), pain and discomfort (30%), dysphagia (26.7%), and hoarseness (20%) were the most common symptoms, with 43.3% of patients being asymptomatic [3]. The variability in symptoms between studies may be due to the different characteristics of the study populations.

The RSG definition is subject to debate, with various definitions remaining subjective [2]. Huins et al. proposed a criterion that includes goiter extent below the thoracic inlet (more than 3 cm), goiter proportion within the breast (at least 50%), and gland extension into the mediastinum below the thoracic inlet. Such scoring systems have been reported in previous research and were used in this study [2,8]. However, a recent analysis of the RSG classifications for their ability to predict intraoperative and postoperative complications found that most definitions of the RSG classifications were not clinically relevant. In addition, Katlic's classification (thyroid with part remaining permanently retrosternal) was most useful in predicting a possible sternotomy to remove the goiter [14].

Except for one case that required a sternotomy, all patients in this study underwent a cervical approach without requiring additional incisions or sternotomies. After requiring a complete sternotomy for treatment, the patient was diagnosed with congenital ectopic intrathoracic thyroid hypertrophy. The methodology we used is consistent with previous studies [1-3]. The majority of surgeons recommend a cervical approach and believe that in up to 98% of cases, this is sufficient to move the goiter into the neck without significant morbidity [13,15]. Video-assisted thoracoscopic surgery and robot-assisted thoracoscopic surgery are becoming increasingly popular in thoracic surgery due to their faster recovery times, less pain, and shorter hospital stays compared to open approaches [16,17]. However, there are few reports of minimally invasive procedures for the removal of posterior mediastinal and ectopic intrathoracic goiters [18,19]. Furthermore, the use of novel procedures requires specialized equipment, making them less suitable for emergency conversion. A meta-analysis reveals that 6.12% of RSG surgeries require extracervical approaches, with risk factors like recurrent goiter, primary mediastinal goiter, and invasive cancer. However, this meta-analysis mentioned that inconsistent literature and smaller series offer lower-quality information, hindering the creation of evidence-based indicators for extracervical methods [16].

In this study, postoperative complications occurred in 22 (31.9%) cases, transient hypocalcemia in 11 (15.9%), permanent hypocalcemia in two (2.9%), transient hypercalcemia in two (2.9%), transient hoarseness in eight (11.6%), postoperative hematoma in two (2.9%), local infection in one (1.4%), and seroma collection in one (1.4%) case. Rugiu et al. found transient hypocalcemia to be the most prevalent issue, aligning with our research findings [20]. In Abdelrahman et al., tracheomalacia (13.4%), transient hypocalcemia (10%), and hypoparathyroidism (6.7%) were the most common complications after thyroidectomy [3]. In another study, postoperative complications occurred in 24 (34.2%) cases (22.2% of patients experienced postoperative hypoparathyroidism, 11.4% developed unilateral laryngeal nerve injury, and 4.2% developed bilateral nerve injury requiring a posterior cordotomy) [21]. Overall, the complication rate in our series was comparable to previous reports [22,23]. This makes sense considering that most cases were found in the anterior mediastinum, where anatomical disruption of the recurrent laryngeal nerve or parathyroid glands is unlikely to occur, both of which occur in the tracheoesophageal groove. On the other hand, there may be a higher risk of injury with posterior extensions that alter the course of the nerve.

In this series, the final histologic diagnosis yielded a malignancy rate of 32%. The literature reports a malignancy rate between 0% and 24.3%, with papillary carcinoma being the most common histological subtype [21]. The rate reported in this study was significantly higher in several series [1,3], as we have shown in previous studies [2].

In this study, advanced age, difficulty swallowing, tracheal deviation, large RSG mass, advanced RSG grade, previous surgery, and malignant histopathology were associated with postoperative complications and were statistically significant. Ayache et al. found that postoperative complications of RSG in 117 thyroidectomies were related to the volume and history of thyroid surgery. Five unilateral RNPs occurred, two remained permanent, six cases had permanent hypocalcemia, and three bleeding cases required reoperation [24]. Van Slycke et al. found that 3.1% of thyroidectomy patients had long-standing hypocalcemia and 1.8% had recurrent laryngeal palsy, with younger age, female gender, and malignancy being risk factors for persistent hypocalcemia. However, no clear risk factors for permanent nerve palsy could be identified. In addition, the female gender, high BMI, and heavier thyroids protected against bleeding [25]. A study of 830 patients found that retrosternal extension and thyroid mass larger than 100 ml were significant factors for temporary and permanent nerve paralysis. Additionally, thyroid cancer was associated with an increase in permanent paralysis but no impact on temporary paralysis. Furthermore, obese patients older than 65 years have doubled the risk of temporary complications, with men more likely to develop phonation disorders. Secondary surgery, particularly thyroidectomy with visual RLN detection, increases vocal cord paralysis risk [26]. The predictive factors for postoperative complications in Li et al. and Bove et al. were recurrences and spread beyond the carina [8,27]. The research by Ghabisha et al. identified risk factors for postoperative complications, including older age, thyroid mass, extension below the aortic arch (grade 2), longer operative time, bleeding, ICU hospitalization, and malignant features [2].

Study limitations

The study results have several limitations, including a retrospective design that limits data collection, a small sample size that may limit statistical power, a lack of clear definitions or criteria for RSG, and insufficient data on family history or previous medical illnesses. These limitations make it difficult to draw sound conclusions and generalize the results to broader populations. Because of the lack of representative samples and the low accuracy of the FNAC method, malignant RSG may not have been detected preoperatively. Furthermore, our study's findings may be influenced by variations in retrosternal grade since we did not have any RSG below the right atrium (grade 3), which could serve as an unnoticed confounding factor in our findings. This limitation may be due to data scarcity and patient selection variability. However, to the best of our knowledge, no study has properly accounted for these characteristics in terms of case size, extension, or presentation specificity. To mitigate these limitations and provide more robust results, we recommend conducting a prospective study with a larger sample and longer follow-up.

## Conclusions

RSG is a rare disease that may require challenging surgical intervention. In this study, the cervical approach was the most practical and least intrusive surgical method. In addition, postoperative complications were associated with advanced age, difficulty swallowing, tracheal deviation, large RSG mass, advanced RSG grade, previous surgery, and malignant histopathology. Low rates of postoperative complications can be attained by understanding the surgical architecture of the neck, essential clinical RSG presentation, thyroid pathology, and necessary surgical treatment. Additionally, the treating surgeon should be conversant with the anatomic categorization and grading of RSG. Furthermore, a large multicenter investigation with more cases is required to obtain agreement on the definition and categorization system.
